# Comparison of the Effects of Multiple Frailty and Nutritional Indexes on Postoperative Outcomes in Critically Ill Patients Undergoing Lung Transplantation

**DOI:** 10.3390/medicina60071018

**Published:** 2024-06-21

**Authors:** Sang-Wook Lee, Donghee Lee, Dae-Kee Choi

**Affiliations:** Department of Anesthesiology and Pain Medicine, Asan Medical Center, University of Ulsan College of Medicine, Seoul 05505, Republic of Korea; sangwooklee20@gmail.com (S.-W.L.); d220404@amc.seoul.kr (D.L.)

**Keywords:** frailty, nutritional index, postoperative mortality, lung transplantation

## Abstract

*Background and Objective*: Lung transplantation is the only life-extending therapy for end-stage pulmonary disease patients, but its risks necessitate an understanding of outcome predictors, with the frailty index and nutritional status being key assessment tools. This study aims to evaluate the relationship between preoperative frailty and nutritional indexes and the postoperative mortality rate in patients receiving lung transplants, and to determine which measure is a more potent predictor of outcomes. *Materials and Methods:* This study reviewed 185 adults who received lung transplants at a single medical center between January 2013 and May 2023. We primarily focused on postoperative 7-year overall survival. Other outcomes measured were short-term mortalities, acute rejection, kidney complications, infections, and re-transplantation. We compared the predictive abilities of preoperative nutritional and frailty indicators for survival using receiver operating characteristic curve analysis and identified factors affecting survival through regression analyses. *Results:* There were no significant differences in preoperative nutritional indicators between survivors and non-survivors. However, preoperative frailty indicators did differ significantly between these groups. Multivariate analysis revealed that the American Society of Anesthesiologists Class V, clinical frailty scale, and Charlson Comorbidity Index (CCI) were key predictors of 7-year overall survival. Of these, the CCI had the strongest predictive ability with an area under the curve of 0.755, followed by the modified frailty index at 0.731. *Conclusions:* Our study indicates that for critically ill patients undergoing lung transplantation, frailty indexes derived from preoperative patient history and functional autonomy are more effective in forecasting postoperative outcomes, including survival, than indexes related to preoperative nutritional status.

## 1. Introduction

Lung transplantation represents the only therapeutic intervention that can extend the life and enhance the quality of life for patients with end-stage pulmonary diseases [1,2]. However, this procedure has significant risks, making it imperative to understand the predictors of surgical outcomes. Over the years, various assessment methods have been employed to evaluate the condition of patients undergoing lung transplantation. Notably, the frailty index and nutritional status indicators have been recognized as valuable tools for predicting postoperative outcomes [3,4]. These indexes provide comprehensive insights into the patient’s physical fitness, immunity, and nutritional health, furnishing reliable information on how a patient might withstand the stresses of surgery and postoperative recovery. Importantly, previous studies have demonstrated that preoperative frailty in lung transplant patients is a significant prognostic factor affecting postoperative outcomes [4,5,6]. A study by Kim et al. (2019) highlighted the significance of the Prognostic Nutritional Index (PNI) as a tool to identify higher-risk lung transplant recipients, suggesting that assessing patients using the PNI preoperatively might aid in decreasing postoperative complications and mortality rates [3]. However, while the potential of the PNI as a predictor of postoperative outcomes has been highlighted, there is a paucity of research delving into its relationship with other frailty scores and nutritional indicators. Comparative studies on the relative efficacy of these indexes are scant, and evidence about their predictive power for specific disease conditions remains limited. Against this backdrop, our research aimed to elucidate the associations between frailty indexes, nutritional status indicators, and postoperative outcomes in patients undergoing lung transplantation. We hypothesized that preoperative frailty index and nutritional status indicators might show a significant correlation with postoperative mortality rates. The goals of this study were to assess the association between preoperative frailty and nutritional status indexes and postoperative mortality rates in lung transplant recipients, and to determine which indicator acts as a stronger predictor of outcomes. Through this, we aimed to derive and suggest more effective indexes for preoperative assessment and management. The findings from our study will offer pivotal insights for the preoperative assessment and management of patients undergoing lung transplantation and may yield novel criteria for predicting future outcomes.

## 2. Methods

### 2.1. Study Design

This study retrospectively analyzed 185 adult patients aged 18 years and older who underwent lung transplantation at Asan Medical Center from January 2013 to May 2023. The research was approved by the Institutional Review Board of Asan Medical Center (Seoul, Korea, approval number 2023-0929, approval date 26 July 2023) and was conducted in accordance with the guidelines of the Declaration of Helsinki. Due to the retrospective nature of the data collection and analysis, the requirement to obtain informed consent from the patients was waived.

### 2.2. Patients

**Figure 1:**
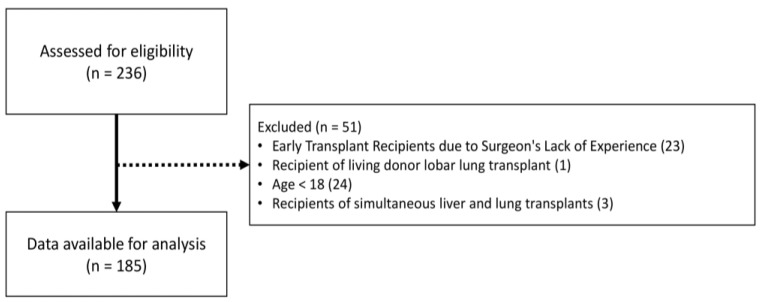
Flow diagram of patient selection.

From the inception of lung transplantation surgeries at Asan Medical Center up until May 2023, out of a total of 236 patients who underwent transplantation, 23 patients underwent early-stage transplant and experienced a poor prognosis due to the surgeon’s lack of experience and thus were excluded from the study. Patients who underwent living-donor lobar lung transplantation were excluded. One patient who underwent living-donor lobar lung transplantation, a total of 24 pediatric patients younger than 18 years, and 3 patients who received simultaneous liver and lung transplantation were excluded from the analysis (Figure 1). In addition, patients for whom follow-up visits were missing and for whom the final clinical outcomes could not be determined were also excluded.

### 2.3. Preoperative Variables and Data Collection

All study data were extracted including preoperative demographic data, preoperative examination data, and postoperative outcome data from the hospital’s electronic medical record (EMR) systems. Information such as demographic data including age, sex, and body mass index (BMI), American Society of Anesthesiologists (ASA) Physical Status classification, type of lung transplantation, smoking status, history of idiopathic pulmonary fibrosis (IPF), diabetes mellitus (DM), or coronary disease, and preoperative use of home O_2_ were extracted. Preoperative blood test results, such as albumin, creatinine, E-lymphocyte, white blood cell count, C-reactive protein, brain natriuretic peptide, and total cholesterol, were evaluated. In addition, preoperative pulmonary function test findings, including the forced expiratory volume in one second (FEV1), forced vital capacity (FVC), and diffusing capacity for carbon monoxide (DLCO), as well as the 6 min walk distance were assessed. Furthermore, the preoperative use of mechanical ventilation, insertion of extracorporeal membrane oxygenation (ECMO) prior to surgery, and intraoperative use of cardiopulmonary bypass (CPB) were also investigated.

### 2.4. Preoperative Nutritional Status and Frailty Risk Indexes

The preoperative nutritional status of patients undergoing transplantation was assessed using the PNI, Geriatric Nutritional Risk Index (GNRI), and Controlling Nutritional Status (CONUT) score [7,8,9,10].

- The PNI can be calculated as follows: PNI = 10 × serum albumin (g/dL) + 0.005 × total lymphocyte count (cells/µL) [8,10].
- The GNRI can be determined by the following equation: GNRI = 14.89 × serum albumin (g/dL) + 41.7 × (present weight/ideal body weight) [7].
- The CONUT score is computed by summing the scores of the following variables:
  1. Serum albumin level: ≥3.5 g/dL (0 points), 3.0–3.4 g/dL (2 points), 2.5–2.9 g/dL (4 points), or <2.5 g/dL (6 points);
  2. Total lymphocyte count: ≥1600 cells/µL (0 points), 1200–1599 cells/µL (1 point), 800–1199 cells/µL (2 points), or <800 cells/µL (3 points);
  3. Total cholesterol level: ≥180 mg/dL (0 points), 140–179 mg/dL (1 point), 100–139 mg/dL (2 points), or <100 mg/dL (3 points) [9].

The preoperative frailty status of patients undergoing transplantation was measured using the Modified Frailty Index (MFI), Clinical Frailty Score (CFS), and Charlson Comorbidity Index (CCI) by reviewing the patients’ medical history recorded in their EMR charts.

- The MFI is calculated based on a patient’s medical history and functional status. It comprises 11 elements, and the score is derived by summing the applicable factors and then dividing by 11. Thus, having one factor corresponds to a score of 0.09, while having three factors results in a score of 0.27 (Supplementary Table S1) [11,12,13].
- The CFS is categorized on a scale ranging from 1 (very fit) to 9 (terminally ill). The score is determined by selecting the fitness level that matches the descriptive criteria provided for each level (Supplementary Table S2) [14].
- The CCI is determined by summing the scores associated with a patient’s medical history to achieve the final score (Supplementary Table S3) [15].

### 2.5. Primary and Secondary Postoperative Outcomes

In our study, the primary postoperative outcome was 7-year overall survival. Secondary outcomes included postoperative mortality within 30 days, postoperative mortality within 90 days, acute rejection, postoperative continuous renal replacement therapy (CRRT) application, postoperative acute kidney injury (AKI), respiratory infection, sepsis, and re-transplantation. Postoperative AKI was defined as an increase in serum creatinine levels by more than 0.3 mg/dL or an increase to over 1.5 times the baseline within 48 h after surgery [16]. Acute rejection was characterized as clinical scenarios in which transplant rejection was suspected and required steroid pulse therapy or when there were pathological findings indicative of rejection. Respiratory infection was defined by the presence of pneumonia findings on postoperative chest radiographs and objective evidence of an infection that required treatment as confirmed by other tests such as sputum culture.

### 2.6. Statistical Analysis

Continuous data are expressed as the mean and standard deviation, and categorical data are denoted as numbers and percentages. When comparing continuous variables between survivors and non-survivors, Student’s *t*-test or the Mann–Whitney U test was employed. To compare categorical variables between groups, the chi-squared test or Fisher’s exact test was used. The predictive performance of various preoperative nutritional and frailty indexes for 7-year overall survival was compared using receiver operating characteristic (ROC) curve analysis. Survival curves were plotted and compared using Kaplan–Meier survival analysis based on the distribution of the index values. Through univariate and multivariate Cox proportional hazard regression analyses, we identified various factors influencing 7-year overall survival. The hazard ratios are presented with 95% confidence intervals. In every analysis, a *p*-value below 0.05 was deemed to indicate statistical significance. All statistical analyses were conducted using the R language (R ver. 4.3.1, R Foundation for Statistical Computing, Vienna, Austria).

## 3. Results

### 3.1. Baseline Patient Characteristics

Table 1 compares the baseline characteristics of the patients who underwent lung transplantation based on their 7-year overall survivor status. Of the 185 patients, 55 (29.7%) died. Among lung transplant recipients, there was no statistically significant difference in age or gender between survivors and non-survivors. Most patients (177, 95.7%) underwent bilateral lung transplantation, while the remaining patients (8, 4.3%) received heart–lung transplantation, and the most common cause of transplantation was IPF. A total of 112 (60.5%) patients were on home O_2_ before transplantation, and there was no statistically significant difference in the proportion of patients who received mechanical ventilation or ECMO preoperatively between survivors and non-survivors. There were no statistically significant differences in the preoperative pulmonary function test results between survivors and non-survivors. However, CPB was applied statistically significantly more commonly during surgery in the non-survivor group than in the survivor group (*p* = 0.004). In addition, the proportion of patients with an ASA class of five or higher, indicating high risk, was statistically significantly greater in the non-survivor group than in the survivor group. Preoperative nutritional status indexes (PNI, GNRI, CONUT) showed no statistically significant difference between survivors and non-survivors. In contrast, the preoperative frailty indexes (MFI, CFS, CCI) were statistically significantly different between the two groups (*p* < 0.05).

### 3.2. Comparing Nutritional and Frailty Indexes in Predicting in-Hospital Mortality

**Figure 2:**
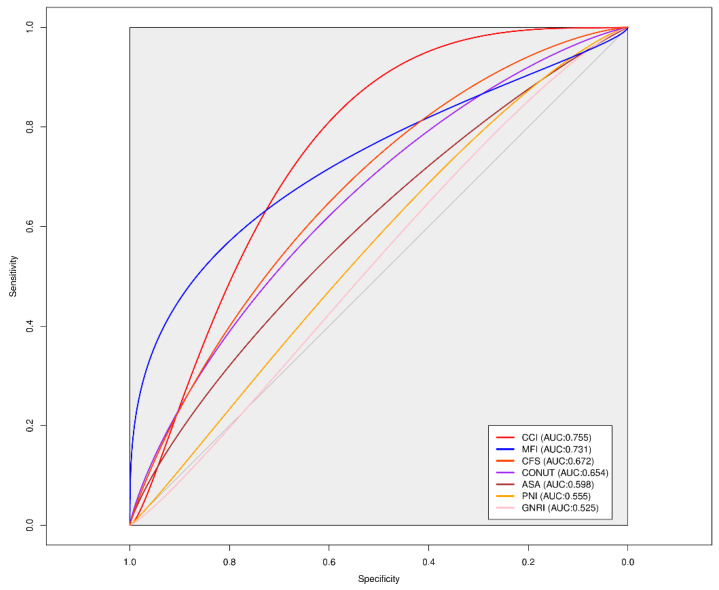
Comparison of ROC curves for frailty scores and preoperative nutritional indexes in predicting 30 days mortality after lung transplantation. PNI, Prognostic Nutritional Index; GNRI, Geriatric Nutritional Risk Index; CONUT, Controlling Nutritional Status; MFI, Modified Frailty Index; CFS, Clinical Frailty Scale; CCI, Charlson Comorbidity Index.

**Figure 3:**
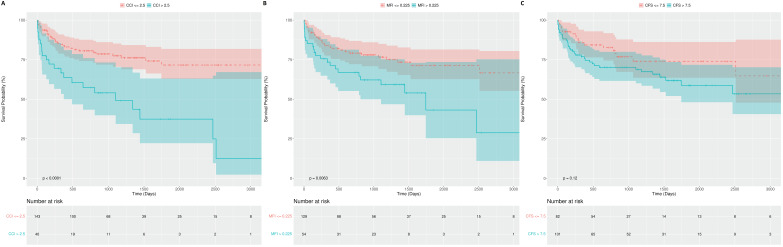
Comparative Kaplan–Meier survival curves of the (**A**) CCI, (**B**) MFI, and (**C**) CFS in patients after lung transplantation.

ROC curve analysis was used to compare the predictive power of preoperative nutritional indexes and frailty indexes in predicting in-hospital mortality within 30 days after surgery. The CCI showed the highest predictive power with an AUC value of 0.755, followed by the MFI with an AUC of 0.731 (Figure 2). Overall, frailty indexes demonstrated better predictive performance than nutritional indexes (PNI, GNRI, CONUT, and albumin) (Figure 2). Through the ROC curve analysis, the median cut-off value for the MFI, determined by Youden’s index, was 0.225. For the CFS, the median cut-off value was 7.5, and for the CCI, it was 2.5. By plotting the Kaplan–Meier survival curves and comparing them based on the cut-off values of each frailty index, we observed a difference in survival rates (Figure 3).

### 3.3. Postoperative Clinical Outcomes

A total of 10 patients (5.4%) died within 30 days after the surgery, and 19 patients (10.3%) died within 90 days (Table 2). There was no statistically significant difference between survivors and non-survivors in terms of secondary postoperative outcomes such as acute rejection, respiratory infection, and re-transplantation. However, the incidence rates of postoperative CRRT, postoperative AKI, and sepsis were statistically significantly higher among non-survivors than among survivors (*p* < 0.001). We examined if the clinical outcomes were distributed differently between the two groups based on the cut-off values of the frailty risk scores and if these differences were statistically significant (Supplementary Table S4). Based on the MFI with a cut-off value of 0.225, the following outcomes were statistically significantly worse in the high MFI group: respiratory infection, 30-day mortality, and overall death (Supplementary Table S4A). Based on the CFS with a cut-off value of 7.5, postoperative CRRT was performed at a statistically significant higher rate in the group with a CFS greater than 7.5 (Supplementary Table S4B). Using a cut-off value of 2.5, the high CCI group had a statistically significantly higher incidence of postoperative CCRT and sepsis (Supplementary Table S4C). In addition, the rates of 30-day postoperative mortality, 90-day mortality, and overall death were significantly higher in this group (Supplementary Table S4C).

### 3.4. Factors Affecting Postoperative 7-Year Overall Survival

Through univariate Cox proportional hazard analysis, among various factors affecting the 7-year overall survival of patients undergoing lung transplantation, ASA Class V, the MFI, the CFS, and the CCI were statistically significantly associated with an increased risk (Table 3). When the statistical significance level was set at 0.1 and only significant variables were considered, subsequent multivariate analysis revealed that ASA Class V, the CFS, and the CCI were ultimately independent prognostic factors influencing 7-year overall survival (Table 3).

## 4. Discussion

In our study, we sought to determine how preoperative nutritional indicators and previously recognized frailty indexes affect postoperative outcomes in patients undergoing lung transplantation. This study demonstrated that frailty indexes are more useful prognostic factors than nutritional status indexes in predicting postoperative 7-year overall survival in patients undergoing lung transplantation.

Previous studies have reported that age, underlying disease, bilateral lung transplantation, and BMI are factors that affect the prognosis of patients undergoing lung transplantation [17,18,19]. However, in our study, the effects of these factors on 7-year overall survival after surgery were not statistically significant. It is thought that these factors may have different effects on outcomes depending on the distribution of the patient population studied. We also believe that these findings from our study are not conclusive and will require further validation through more multicenter data analyses.

Kim et al. have suggested that the preoperative PNI score is a prognosticator of postoperative outcome in patients undergoing lung transplantation [3]. However, in our study, neither the PNI nor other nutritional markers such as the GNRI or CONUT score showed a statistically significant association with postoperative 7-year overall survival. This lack of association of nutritional status indicators with postoperative prognosis, as compared with the findings of a previous study by Kim et al., is likely due to the different preoperative nutritional statuses of the patients in the study. In the previous study, the patients were relatively well-nourished with a mean albumin level of 3.5, whereas the mean albumin level of the patients in this study was lower at 2.9. This may be due to the fact that the patients in the previous study had a good preoperative nutritional status; therefore, a deterioration in this metric would have had a greater impact on prognosis. In contrast, the preoperative nutritional status of the patients in this study was already poor for most of the patients; thus, the impact of preoperative nutritional status on postoperative prognosis may have been limited.

In patients undergoing lung transplantation, preoperative frailty is known to be a major predictor of postoperative morbidity and mortality [5,6]. Prior studies in patients undergoing lung transplantation have mostly used preoperative frailty measures based on the Short Physical Performance Battery and the Fried Frailty Phenotype [5,20,21]. Most of these metrics require specific patient measurements such as grip strength, gait speed, chair stands, and balance [20,21]. However, it is unrealistic to expect all patients undergoing lung transplantation to undergo these measurements before surgery. It is not practical to apply these frailty measures to patients who are sedated with mechanical ventilation in the intensive care unit or who are unconscious. Furthermore, patients who are able to have their gait speed, grip strength, balance, and chair stands measured prior to surgery are likely to be less frail than those who are unable to, and selecting only these patients for inclusion in this study would likely have introduced selection bias; therefore, only mildly frail patients were included. Therefore, we chose the MFI and CFS, which are assessed based on the patient’s history and functional independence, from a broader range of frailty measures applicable to all patients undergoing lung transplantation. A major advantage of these frailty indexes is that they can be used to measure a patient’s preoperative frailty through a chart review using only information that can be automatically extracted from EMRs for clinical application. No previous studies of frailty measures such as the MFI or CFS have been conducted in patients undergoing lung transplantation, and how useful these measures could be in real-world clinical practice is not well understood. The performance of these frailty indicators as predictors of postoperative prognosis was not inferior to that of frailty indicators used in previous studies based on C-statistics, making them very convenient to apply in real clinical practice and applicable to a wide range of patients undergoing lung transplantation, including unconscious patients. Our study demonstrated that these frailty indicators are better at predicting postoperative outcomes, such as postoperative mortality, than preoperative nutritional indicators. We have shown that, at least in patients undergoing lung transplantation, these frailty indicators may be more informative than nutritional indicators in prioritizing transplantation as well as postoperative mortality. Therefore, in patients undergoing lung transplantation, these frailty indicators should be considered in conjunction with preoperative nutritional indicators to predict postoperative prognosis and prioritize transplantation. 

Our study is the first to compare the performance of various nutritional and frailty markers for predicting postoperative prognosis in patients undergoing lung transplantation. Therefore, we believe that this is a very valuable study to compare the usefulness of these metrics as predictors of postoperative outcomes and to determine which metrics are appropriate for application in patients undergoing lung transplantation.

Our study has several limitations. First, we analyzed all data retrospectively. Therefore, we believe that future prospective studies should be conducted to validate our findings. Second, while the number of patients in our study may seem small, it is substantial when compared with the data from previously reported studies on patients undergoing lung transplantation in Asian countries. However, further studies should be conducted with a larger number of patients to validate the findings of our study. Finally, this study is based on data from a single institution. Notably, our data included a higher proportion of moribund patients requiring perioperative mechanical ventilation than previous studies, so it may be difficult to generalize our findings to all patients at other institutions. However, our findings may be applicable to critically ill patients, especially those who are on mechanical ventilation prior to surgery, such as the patients in our study. Future studies with multicenter or national data will be needed to confirm the findings.

## 5. Conclusions

Our study suggests that, in patients undergoing lung transplantation, frailty indexes based on preoperative patient history and functional independence are valuable prognostic factors for predicting various postoperative outcomes, including postoperative survival. These indexes are more informative compared to those reflecting preoperative nutritional status, which have zero predictive value. Furthermore, these prognostic factors are no less effective than traditional frailty prognostic factors that require specific frailty measurement tests. In particular, these findings could prove valuable for the management of critically ill patients who require mechanical ventilation or who are unconscious prior to undergoing surgery. Additionally, preoperative frailty assessment using frailty indices in critically ill recipients undergoing lung transplantation will enable the identification of high-risk patients and contribute to the efficient allocation of hospital resources. However, it is essential to note that these results require validation through retrospective or prospective studies with larger, multicenter datasets. Further prospective research is warranted to determine whether preoperative rehabilitation programs or frailty-enhancing treatments can enhance postoperative outcomes when selecting high-risk patients using these preoperative frailty measures.

## Figures and Tables

**Table 1:** Baseline characteristics of recipients who underwent lung transplantation according to overall survival status.

Variables	Total (*n* = 185)	Survivors (*n* = 130)	Non-Survivors (*n* = 55)	*p*-Value
Age, years	53.9 ± 11.6	53.4 ± 11.2	55.2 ± 12.5	0.082
Male sex, *n* (%)	119 (64.3)	83 (63.8)	36 (65.5)	0.967
BMI, kg/m^2^	22.0 ± 4.3	21.7 ± 4.0	22.6 ± 5.1	0.356
ASA Class III, *n* (%)	55 (29.7)	41 (31.5)	14 (25.5)	0.041
ASA Class VI, *n* (%)	121 (65.4)	86 (66.2)	35 (63.6)	
ASA Class V, *n* (%)	9 (4.9)	3 (2.3)	6 (10.9)	
Bilateral lung transplantation, *n* (%)	177 (95.7)	124 (95.4)	53 (96.4)	1.000
Former or current smoker, *n* (%)	96 (51.9)	68 (52.3)	28 (50.9)	0.990
Underlying disease				
IPF, *n* (%)	117 (63.2)	80 (61.5)	37 (67.3)	0.567
DM, *n* (%)	36 (19.5)	24 (18.5)	12 (21.8)	0.746
Coronary disease, *n* (%)	29 (15.7)	19 (14.6)	10 (18.2)	0.698
Home O_2_, *n* (%)	112 (60.5)	74 (56.9)	38 (69.1)	0.167
Creatinine, mg/dL	0.63 ± 0.27	0.61 ± 0.26	0.68 ± 0.30	0.189
Albumin, mg/dL	2.91 ± 0.81	2.94 ± 0.88	2.86 ± 0.61	0.877
E-lymphocyte, /mm^3^	13.57 ± 7.93	13.20 ± 8.11	14.45 ± 7.50	0.203
White blood cell count, 10^3^/µL	11.17 ± 4.42	11.83 ± 4.58	9.62 ± 3.59	0.002
C-reactive protein, mg/dL	5.65 ± 5.71	5.83 ± 5.86	5.23 ± 5.37	0.641
Brain natriuretic peptide, pg/mL	301.6 ± 544.3	260.3 ± 400.8	389.6 ± 763.1	0.075
Total cholesterol, mg/dL	145.5 ± 46.0	146.7 ± 45.6	142.3 ± 47.2	0.572
Pretransplantation PFT				
FEV1, predicted (%)	44.7 ± 17.7	43.1 ± 18.2	48.5 ± 16.1	0.090
FVC, predicted (%)	45.1 ± 16.1	44.2 ± 16.8	46.9 ± 14.2	0.180
DLCO, predicted (%)	25.1 ± 13.7	25.0 ± 13.5	25.5 ± 14.4	0.987
6-Minute walk distance, m	238.2 ± 116.3	244.7 ± 119.2	223.4 ± 109.7	0.354
Preoperative MV, *n* (%)	128 (69.2)	87 (66.9)	41 (74.5)	0.394
Preoperative ECMO, *n* (%)	108 (58.4)	73 (56.2)	35 (63.6)	0.435
Intraoperative CPB, *n* (%)	73 (39.5)	42 (32.3)	31 (56.4)	0.004
PNI	36.1 ± 9.8	36.5 ± 10.4	35.2 ± 8.5	0.476
GNRI	84.5 ± 14.9	84.3 ± 15.5	84.9 ± 13.4	0.596
CONUT score	6.04 ± 3.15	5.89 ± 3.09	6.40 ± 3.29	0.299
MFI	0.18 ± 0.10	0.17 ± 0.10	0.21 ± 0.10	0.016
CFS	7.46 ± 1.59	7.25 ± 1.69	7.96 ± 1.22	0.012
CCI	1.84 ± 1.26	1.70 ± 1.24	2.18 ± 1.26	0.002

Values are presented as the mean ± standard deviation or number (percentage). BMI, body mass index; ASA, American Society of Anesthesiologists; IPF, idiopathic pulmonary fibrosis; DM, diabetes mellitus; PFT, pulmonary function test; MV, mechanical ventilation; ECMO, extracorporeal membrane oxygenation; CPB, cardiopulmonary bypass; PNI, Prognostic Nutritional Index; GNRI, Geriatric Nutritional Risk Index; CONUT, Controlling Nutritional Status; MFI, Modified Frailty Index; CFS, Clinical Frailty Scale; CCI, Charlson Comorbidity Index.

**Table 2:** Postoperative clinical outcomes among lung transplant recipients according to overall survival status.

Outcomes	Total (*n* = 185)	Survivors (*n* = 130)	Non-Survivors (*n* = 55)	*p*-Value
Acute rejection, *n* (%)	9 (4.9)	4 (3.1)	5 (9.1)	0.129
Postoperative CRRT, *n* (%)	27 (14.6)	6 (4.6)	21 (38.2)	<0.001
Postoperative AKI, *n* (%)	43 (23.2)	16 (12.3)	27 (49.1)	<0.001
Respiratory infection, *n* (%)	69 (37.3)	43 (33.1)	26 (47.3)	0.097
Sepsis, *n* (%)	40 (21.6)	15 (11.5)	25 (45.5)	<0.001
Re-transplantation, *n* (%)	2 (1.1)	1 (0.8)	1 (0.8)	0.507
30-day mortality, *n* (%)	10 (5.4)			
90-day mortality, *n* (%)	19 (10.3)			

CRRT, continuous renal replacement therapy; AKI, acute kidney injury.

**Table 3:** Univariate and multivariate Cox proportional hazards analysis of various factors influencing 7-year overall survival.

Variables	Univariate		Multivariate	
	HR (95% CI)	*p*-Value	HR (95% CI)	*p*-Value
Age, years	1.022 (0.993–1.051)	0.137		
Male sex	1.037 (0.597–1.800)	0.899		
BMI	1.037 (0.960–1.119)	0.355		
ASA Class III	Reference		Reference	
ASA Class VI	1.212 (0.644–2.283)	0.551	0.780 (0.383–1.588)	0.493
ASA Class V	3.626 (1.428–9.209)	0.007	2.932 (1.202–7.149)	0.018
Former or current smoker	0.982 (0.579–1.666)	0.947		
IPF	1.361 (0.769–2.410)	0.290		
DM	1.498 (0.793–2.828)	0.213		
Coronary disease	1.651 (0.861–3.164)	0.131		
Home O_2_	1.523 (0.856–2.709)	0.152		
Albumin	0.928 (0.674–1.277)	0.646		
C-Reactive Protein	0.986 (0.939–1.036)	0.572		
Brain natriuretic peptide	1.000 (1.000–1.001)	0.082	1.000 (1.000–1.001)	0.230
6-Minute walk distance	0.999 (0.996–1.002)	0.512		
Preoperative MV	1.330 (0.729–2.428)	0.353		
Preoperative ECMO	1.286 (0.749–2.208)	0.362		
Intraoperative CPB	1.672 (0.970–2.881)	0.064	1.591 (0.879–2.880)	0.125
PNI	0.989 (0.962–1.017)	0.448		
GNRI	1.002 (0.985–1.019)	0.810		
CONUT score	1.048 (0.959–1.145)	0.303		
MFI	25.932 (1.711–393.055)	0.019	5.223 (0.174–156.503)	0.341
CFS	1.203 (1.009–1.435)	0.039	1.211 (1.007–1.455)	0.041
CCI	1.258 (1.077–1.470)	0.004	1.325 (1.092–1.607)	0.004

HR, hazard ratio; CI, confidence interval; BMI, body mass index; ASA, American Society of Anesthesiologists; IPF, idiopathic pulmonary fibrosis; DM, diabetes mellitus; MV, mechanical ventilation; ECMO, extracorporeal membrane oxygenation; CPB, cardiopulmonary bypass; PNI, Prognostic Nutritional Index; GNRI, Geriatric Nutritional Risk Index; CONUT, Controlling Nutritional Status; MFI, Modified Frailty Index; CFS, Clinical Frailty Scale; CCI, Charlson Comorbidity Index.

## Data Availability

The data utilized to support the findings of this research can be obtained from the corresponding author upon inquiry.

## Supplementary Materials

The following supporting information can be downloaded at: https://www.mdpi.com/article/10.3390/medicina60071018/s1, Table S1: Eleven components of the Modified Frailty Index; Table S2: Clinical Frailty Score; Table S3: Charlson Comorbidity Index; Table S4: Postoperative clinical outcomes among lung transplant recipients according to the cut-off values of Modified Frailty Index, Clinical Frailty Scale, and Charlson Comorbidity Index.
