# Supplementary material for: Comparison of the Effects of Multiple Frailty and Nutritional Indexes on Postoperative Outcomes in Critically Ill Patients Undergoing Lung Transplantation
Source: Medicina (Kaunas). 2024 Jun 21;60(7):1018. doi: 10.3390/medicina60071018 (PMC11279017; doi:10.3390/medicina60071018)
Supplement: Supplementary file 1 [file medicina-60-01018-s001.zip › medicina-3038832-supplementary.pdf]

Supplementary Material

Comparison of the effects of multiple frailty and nutritional indexes on postoperative outcomes in critically ill patients undergoing lung transplantation

Sang-Wook Lee, MD PhD[1], Donghee Lee, MD[1], Dae-Kee Choi, MD[1*]

[1]Department of Anesthesiology and Pain Medicine, Asan Medical Center, University of Ulsan College of Medicine, Seoul, Republic of Korea

Contents

Table S1. Eleven components of the Modified Frailty Index.

Table S2. Clinical Frailty Score.

Table S3. Charlson Comorbidity Index.

Table S4. (A) Postoperative clinical outcomes among lung transplant recipients according to the cut-off value of the Modified Frailty Index.

Table S4. (B) Postoperative clinical outcomes among lung transplant recipients according to the cut-off value of the Clinical Frailty Scale.

Table S4. (C) Postoperative clinical outcomes among lung transplant recipients according to the cut-off value of the Charlson Comorbidity Index.

Table S1. 11 Eleven components of the Modified Frailty Index.

Diabetes mellitus
Functional status 2 (not independent)
Congestive heart failure
Myocardial infarction
Hypertension requiring medication
Transient ischemic attack or cerebrovascular accident
Cerebrovascular accident with neurologic deficits
Chronic obstructive pulmonary disease or pneumonia
Previous percutaneous coronary intervention, cardiac surgery, or angina
Peripheral vascular disease
Impaired sensorium

Table S2. Clinical Frailty Score.

Score	Fitness	Description
1	Very Fit	People who are robust, active, energetic, and motivated. These people commonly exercise regularly. They are among the fittest for their age.
2	Well	People who have no active disease symptoms but are less fit than category 1. Often, they exercise or are very active occasionally (e.g., seasonally).
3	Managing Well	People whose medical problems are well controlled but are not regularly active beyond routine walking.
4	Vulnerable	While not dependent on others for daily help, often symptoms limit activities. A Common complaint is being "slowed up', and/or being tired during the day.
5	Mildly Frail	These people often have more evident slowing, and need help in high order IADLs (finances, transportation, heavy housework, medications). Typically, mild frailty progressively impairs shopping and walking outside alone, meal preparation and housework.
6	Moderately Frail	People need help with all outside activities and with keeping house. Inside, they often have problems with stairs and need help with bathing and might need minimal assistance(cuing, standby) with dressing.
7	Severely Frail	Completely dependent for personal care from whatever cause (physical or cognitive). Even so, they seem stable and not at high risk of dying (within ~ 6 months).
8	Very Severely Frail	Completely dependent, approaching the end of life. Typically, they could not recover even from a minor illness.
9	Terminally Ill	Approaching the end of life. This category applies to people with a life expectancy <6 months, who are not otherwise evidently frail.

IADL, Instrumental activities of daily living.

Table S3. Charlson Comorbidity Index.

Comorbidity	Weighted Score
Myocardial infarction	1
Congestive heart failure	1
Peripheral vascular disease	1
Cerebrovascular disease	1
Dementia	1
Chronic pulmonary disease	1
Connective tissue disease	1
Peptic ulcer disease	1
Mild liver disease	1
Diabetes	1
Cerebrovascular event (hemiplegia)	1
Moderate or severe renal disease	1
Diabetes with chronic complications	2
Cancer without metastasis	2
Leukemia	2
Lymphoma	2
Moderate or severe liver disease	3
Metastatic solid tumor	6
Acquired immunodeficiency syndrome (AIDS)	6

Table S4. (A) Postoperative clinical outcomes among lung transplant recipients according to the cut-off value of the Modified Frailty Index.

Outcomes	MFI ≤ 0.225 (n = 130)	MFI > 0.225 (n = 55)	P-value
Acute rejection, n (%)	5 (3.8)	4 (7.3)	0.454
Postoperative CRRT, n (%)	17 (13.1)	10 (18.2)	0.502
Postoperative AKI, n (%)	30 (23.1)	13 (23.6)	1.000
Respiratory infection, n (%)	42 (32.3)	27 (49.1)	0.046
Sepsis, n (%)	29 (22.3)	11 (20.0)	0.878
Re-transplantation, n (%)	1 (0.8)	1 (1.8)	0.507
30-day mortality, n (%)	3 (2.3)	7 (12.7)	0.008
90-day mortality, n (%)	10 (7.7)	9 (16.4)	0.131
Death, n (%)	31 (23.8)	24 (43.6)	0.012

MFI, Modified Frailty Index; CRRT, continuous renal replacement therapy; AKI, acute kidney injury.

Table S4. (B) Postoperative clinical outcomes among lung transplant recipients according to the cut-off value of the Clinical Frailty Scale.

Outcomes	CFS ≤ 7.5 (n = 82)	CFS > 7.5 (n = 103)	P-value
Acute rejection, n (%)	2 (2.4)	7 (6.8)	0.303
Postoperative CRRT, n (%)	6 (7.3)	21 (20.4)	0.022
Postoperative AKI, n (%)	13 (15.9)	30 (29.1)	0.051
Respiratory infection, n (%)	24 (29.3)	45 (43.7)	0.063
Sepsis, n (%)	15 (18.3)	25 (24.3)	0.423
Re-transplantation, n (%)	0 (0)	2 (1.9)	0.504
30-day mortality, n (%)	2 (2.4)	8 (7.8)	0.189
90-day mortality, n (%)	6 (7.3)	13 (12.6)	0.349
Death, n (%)	18 (22.0)	37 (35.9)	0.057

CFS, Clinical Frailty Scale; CRRT, continuous renal replacement therapy; AKI, acute kidney injury.

Table S4. (C) Postoperative clinical outcomes among lung transplant recipients according to the cut-off value of the Charlson Comorbidity Index.

Outcomes	CCI ≤ 2.5 (n = 144)	CCI > 2.5 (n = 41)	P-value
Acute rejection, n (%)	7 (4.9)	2 (4.9)	1.000
Postoperative CRRT, n (%)	13 (9.0)	14 (34.1)	< 0.001
Postoperative AKI, n (%)	30 (20.8)	13 (31.7)	0.213
Respiratory infection, n (%)	48 (33.3)	21 (51.2)	0.057
Sepsis, n (%)	22 (15.3)	18 (43.9)	< 0.001
Re-transplantation, n (%)	1 (0.7)	1 (2.4)	0.395
30-day mortality, n (%)	5 (3.5)	5 (12.2)	0.045
90-day mortality, n (%)	9 (6.2)	10 (24.4)	0.002
Death, n (%)	32 (22.2)	23 (56.1)	< 0.001

CCI, Charlson Comorbidity Index; CRRT, continuous renal replacement therapy; AKI, acute kidney injury.
